# Viabahn-assisted sutureless anastomosis (VASA) repair of a complex internal carotid artery aneurysm

**DOI:** 10.1016/j.jvscit.2023.101161

**Published:** 2023-03-20

**Authors:** Luis H. Arzola, Kevin Mani, Gianmarco Zuccon, Tomas Ekberg, Anders Wanhainen

**Affiliations:** a Department of Surgery, Section of Vascular Surgery and Endovascular Therapy, National Institute of Medical Sciences and Nutrition Salvador Zubirán, Mexico City, Mexico; b Department of Surgical Sciences, Section of Vascular Surgery, Uppsala University, Uppsala, Sweden; c Department of Otorhinolaryngology-Head and Neck Surgery, Uppsala University Hospital, Uppsala, Sweden; d Department of Surgical and Perioperative Sciences, Surgery, Umeå University, Umeå, Sweden

**Keywords:** Anastomosis, Carotid artery aneurysm, Covered self-expandable stent, GORE Viabahn, Sutureless

## Abstract

Extracranial carotid artery aneurysms (CAAs) are extremely rare and often require surgical intervention to avoid complications such as local compression symptoms and thrombo-embolization. We present the case of a 63-year-old man with a history of hypertension, meningioma, and an incidental finding of a right saccular internal carotid artery aneurysm at the base of the skull. He underwent open surgical repair; nonetheless, end-to-end anastomosis was not feasible. As bailout, the internal carotid artery was successfully reconstructed with a novel Viabahn-assisted sutureless anastomosis technique (GORE, Viabahn). Postoperative clinical assessment revealed no complications, postoperative computed tomography angiography revealed a patent reconstruction, and the patient was discharged home uneventfully with 1-year clinical and computed tomography angiography follow-up without remarks. Hybrid procedure is a viable option for technically challenging carotid anastomoses near the skull base.

Extracranial carotid artery aneurysms (CAAs) are extremely rare, representing 1% to 4% of all peripheral arterial aneurysms, and can be related mostly to cervical dissection (post-traumatic or spontaneous) but can also occur from atherosclerosis, fibromuscular dysplasia, infection, and post-procedure punctures (iatrogenic).^1^ Frequently extracranial CAAs require surgical intervention to avoid complications such as local compression symptoms and thrombo-embolism.^1^^,^^2^ Open surgery with aneurysmectomy and primary end-to-end anastomosis is the standard of treatment. However, sometimes artery reconstructions near the skull base can be technically very challenging. Being less invasive, the endovascular approach has been considered for high-risk patients but may be hampered by anatomical difficulties, such as small-diameter angulated vessels.^3^ Herein, we describe the case of a man with extracranial CAA at the base of the skull treated successfully with a hybrid technique. The patient has consented to the publication of this manuscript.

## Case report

**Fig 1:**
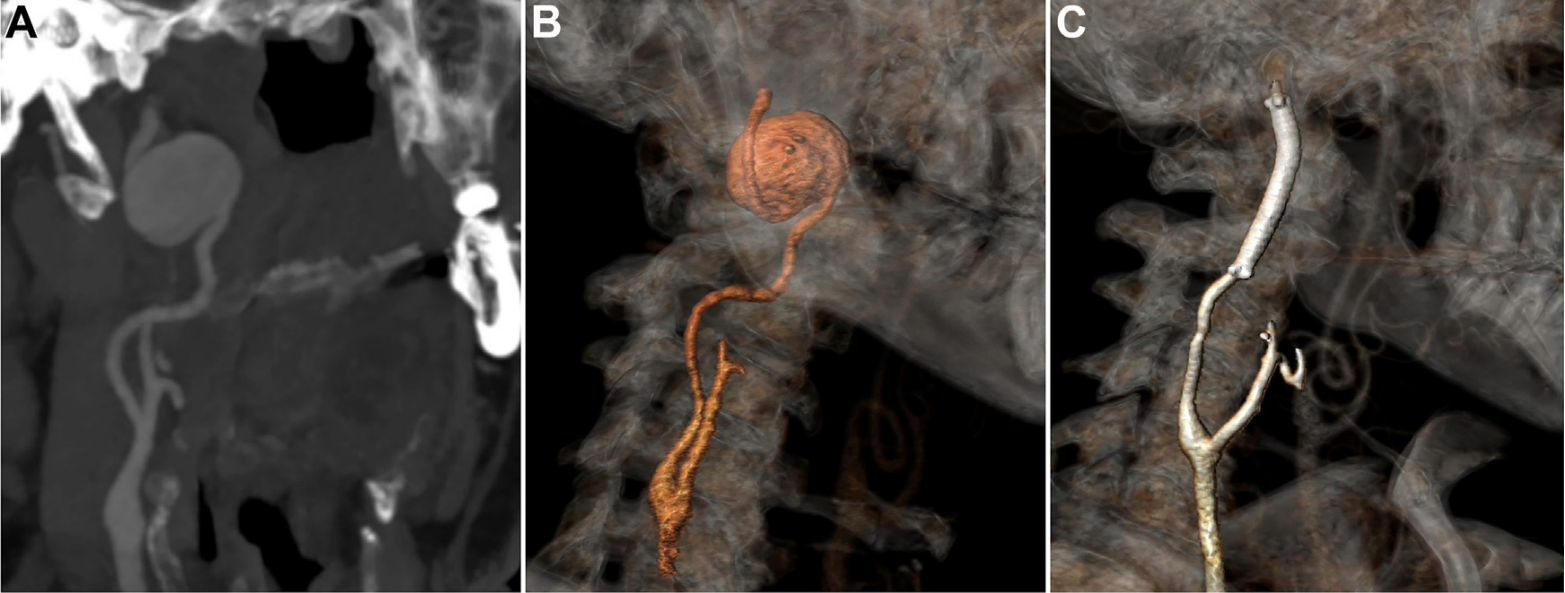
Preoperative computed tomography angiography (CTA) in a coronal view showing a distal saccular extracranial carotid artery aneurysm (CAA) Attigah classification Type I **(A)**. Three-dimensional reconstructions of CTA at admission **(B)** and after Viabahn-assisted sutureless anastomosis (VASA) repair **(C)**.

A 63-year-old man with prior history of hypertension and meningioma was referred to our service due to an incidental finding of a saccular internal CAA. Computed tomography angiography (CTA) revealed a 23-mm right saccular internal CAA near the skull base without thrombus or atherosclerotic plaques (Fig 1). The patient was asymptomatic, and the physical exam demonstrated a palpable pulsatile mass on his right neck. In conjunction with the Head and Neck Surgery service, we planned open surgical repair with extended exposure to the ear and vascular reconstruction with end-to-end anastomosis of the internal carotid artery (ICA).

**Fig 2:**
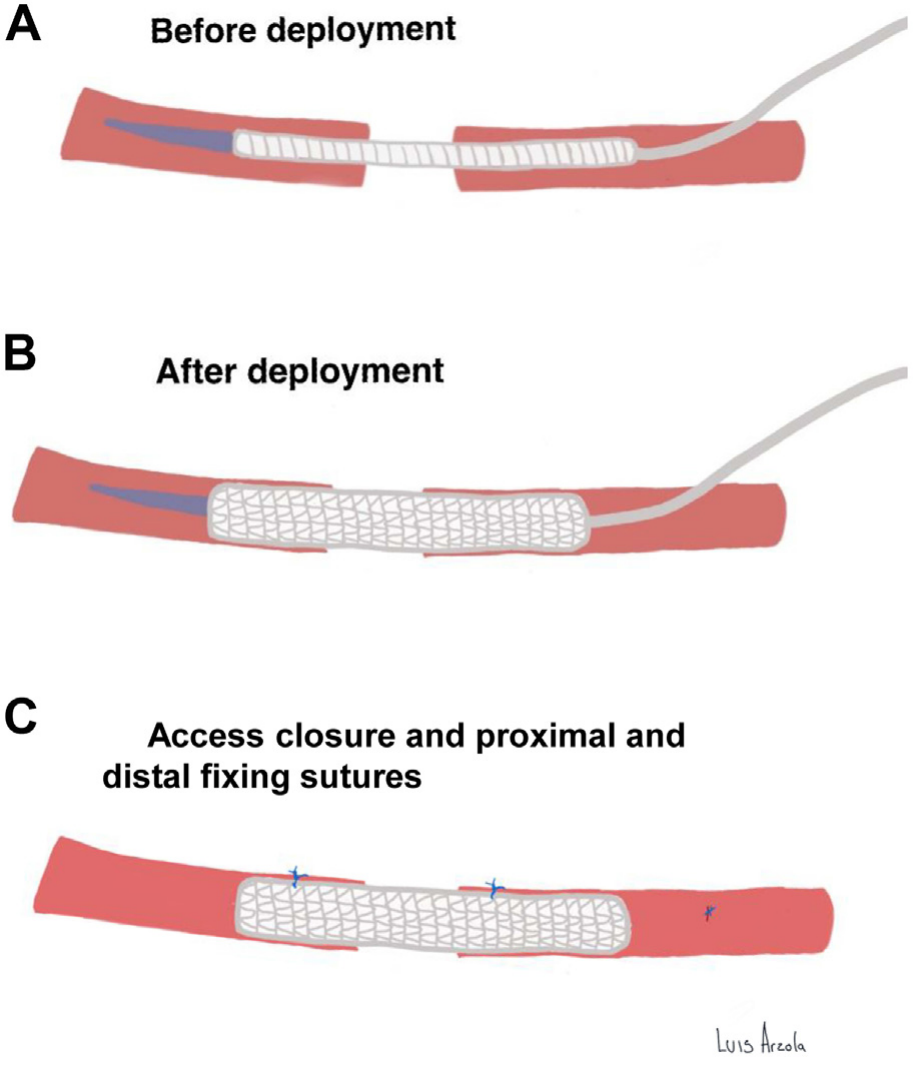
Viabahn-assisted sutureless anastomosis (VASA) technique.

Under general anesthesia and cerebral monitoring, a longitudinal incision from the anterior border of the sternocleidomastoid muscle to the front of the right earlobe was performed. After exposure of the common carotid artery (CCA) and the carotid bifurcation, the hypoglossal nerve was isolated and secured with a vessel loop. The digastric muscle was divided exposing the aneurysm base where the inflow was located antero-caudal and the outflow latero-caudal. The distal portion of the ICA was isolated, and, after administration of systemic heparin, clamped. Attempts at standard end-to-end anastomosis failed due to the small caliber and poor quality of the vessel. After addressing multiple bleeds caused by suture tears, there was insufficient flow, and further surgical attempts were deemed futile. As bailout, we performed a small transverse incision at the proximal part of the ICA from where a 5-mm × 5-cm long covered self-expandable stent (GORE, Viabahn) was inserted to bridge inflow and outflow (Fig 2). The Viabahn was manually introduced under direct visualization into an open (unclamped) distal ICA stump, and without a wire to avoid dissection of the intracranial portion of the ICA. After deployment, patency was confirmed with intraoperative transit-time flow measurement showing over 200 mL/minute of blood flow. Finally, fixing sutures between the stent and the vessel with 6-0 prolene were placed (3 proximal and 1 distal), and the wound was closed in the usual manner.

The patient was moved to the postoperative ward awake and with stable signs. Neurological and otorhinolaryngologic assessment was done at 24 hours with no evidence of acute complications. CTA was performed on postoperative day 3 and showed adequate contrast flow through the stent with a normal filling of the distal vessels. Postoperatively, the patient was put on dual antiplatelet therapy for 3 months; thenceforward, single therapy with aspirin. The patient was discharged home with no complications. One-year clinical and CTA follow-up was without remark. Continued surveillance is planned with duplex annually.

## Discussion

According to de Jong et al, extracranial CAA is defined as a localized dilation greater than 200% of the diameter of the ICA or greater than 150% of the diameter of the CCA.^4^ Extracranial CAAs are more often found in men, with a 49% to 86% predominance reported in previous studies**.** It is reported that true aneurysms are mainly originated after spontaneous carotid artery dissections, whereas false aneurysms are generally related to prior carotid endarterectomy and sometimes infection.^1^^,^^3^^,^^5^ Typically, these patients can present with local obstructive symptoms (dysphagia and dyspnea), a local pulsatile mass, and neurological symptoms arising from thrombo-embolism (amaurosis fugax, transient ischemia attack, stroke), and/or cranial nerve deficit.^2^^,^^6^ The Attigah classification, originally described in 2008, establishes five groups of extracranial CAAs based on anatomy and localization. Type I is an isolated and short aneurysm of the ICA above the carotid bifurcation; type II is a long aneurysm of the ICA (from the carotid bulb to the line of Blaisdell); type III is an aneurysm of the proximal ICA and the carotid bifurcation; type IV is an aneurysm involving both the ICA and CCA distally; and type V is an isolated aneurysm of the CCA.^7^ The most prevalent is the type I (39%), followed by type III (32%).^2^^,^^7^

The potential severe complications of this disease mandate a relatively urgent surgical intervention. Until the 1950s, surgical ligation of the CCA was the standard treatment (described by Sir Astley Cooper in 1808), which had a mortality/major stroke rate of 20% to 40%.^6^ More recently, new surgical techniques have developed and generally include aneurysm resection and end-to-end anastomosis, resection with interposition graft, extracranial to intracranial bypass, aneurysm clipping, and ligation, with a reported mortality/major stroke rate of 2.6% to 9% and a 26% rate of cranial nerve palsy, respectively.^6^^,^^8^ Being less invasive, the endovascular approach has been considered for high-risk patients. Endovascular stenting is technically feasible with a 92.8% success rate and a mortality of 4.1% reported by Li et al^9^ and a lower cranial nerve injury rate.^10^^,^^11^ Sometimes its use may, however, be hampered by anatomical difficulties, such as small diameter and severely angulated vessels associated with extracranial CAA. On the other hand, a systemic review from Paraskevas et al showed that 95% of 166 false aneurysms after carotid dissection either remained unchanged or regressed in size during a mean follow-up of 38 months, suggesting that conservative management and serial surveillance is the optimal treatment for this type of extracranial CAA.^12^^,^^13^

Hybrid repair of the internal carotid artery is a feasible option as reported by Arens et al, with no complications during a 24-month follow-up. They used a GORE hybrid vascular graft prosthetic stent graft as an interposition graft because of a short extracranial stump of the distal ICA near the skull base.^14^ This can also be achieved with the Viabahn open revascularization technique (VORTEC) and the Viabahn Padova sutureless (ViPS) technique, which both are based on telescoping a vascular prosthetic graft in a sutureless fashion with the target vessel by means of a bridging Viabahn stent graft.^15^^,^^16^

Resection back to healthy artery and interposition graft would be the standard in case of technical problems with the anastomosis. In this particular case, we decided to perform the VASA technique due to the combination of a high location of the aneurysm (Attigah Type 1 extracranial CAA) and the frailty and size of the distal ICA stump. This technique discourages the need of suturing a PTFE graft to the proximal stump and dramatically decreases clamping time in urgent or unexpected situations.^17^ We prefer to insert the Viabahn under the supervision of the eye without the support of a guidewire. However, it is important not to forcefully advance the stent graft at the slightest resistance. Although a guidewire will facilitate the advancement of the stent graft into the more angulated intra petrous portion of the vessel, inserting a guidewire blindly into this segment can be risky and should only be done cautiously. In such a situation, one should preferably continue under fluoroscopic guidance, or consider ligating the vessel rather than proceeding with stent grafting into the skull base. The resulting back bleeding from the ICA during the advancement and deployment of the Viabahn is usually manageable, and due to the speed of the method, also limited in duration. A completion angiography should be considered, especially if deployment of the stent graft has caused problems or if the flow is not adequate. In this case, however, with controlled deployment under the supervision of the eye together with good flow, we did not consider it necessary to perform a final angiography. When performed, we prefer to do it through the groin to minimize the trauma to the operated carotid vessel. Endovascular correction of any problems is then often possible, also distally in the ICA.

There are some limitations to this technique. A good landing zone is needed (preferably >2 cm) on both sides to achieve adequate sealing. Additionally, in case of active infection, the usage of the VASA technique is contraindicated.

## Conclusion

Treatment of extracranial CAAs requires an approach determined by size, location, etiology, and comorbidities of these patients. More recently, hybrid procedures consisting of open surgery plus endovascular stenting are pushing further the limits of treatment with excellent short-term results and may be considered as a feasible backup plan in case of anticipated technical or anatomical difficulties. On the other hand, we are still limited by long-term results, so further studies are needed to evaluate patency and complications rates.
